# The effects of combined resveratrol and high intensity interval training on the hippocampus in aged male rats: An investigation into some signaling pathways related to mitochondria

**DOI:** 10.22038/IJBMS.2022.57780.12853

**Published:** 2022-02

**Authors:** Maryam Amirazodi, Amin Mehrabi, Mohammad Amin Rajizadeh, Mohammad Abbas Bejeshk, Khadijeh Esmaeilpour, Farhad Daryanoosh, Abbasali Gaeini

**Affiliations:** 1 Neuroscience Research Center, Institute of Neuropharmacology, Kerman University of Medical Sciences, Kerman, Iran; 2 Shiraz University International Division, Shiraz University, Shiraz, Iran; 3 Department of Exercise Physiology, Kish International Campus, University of Tehran, Kish, Iran; 4 Department of Physiology and Pharmacology, Kerman University of Medical Sciences, Kerman, Iran; 5 Department of Exercise Physiology, Shiraz University, Shiraz, Iran; 6 Department of Exercise Physiology, University of Tehran, Tehran, Iran

**Keywords:** Aging, Hippocampus, HIIT exercise, Oxidative stress, Rat, Resveratrol, Sirtuins

## Abstract

**Objective(s):**

High-intensity interval training (HIIT) is a shape of interval training that provides ameliorated athletic capacity and has a good effect on health. Resveratrol is a natural polyphenol abundant in grapes and red wine and has been demonstrated to apply various useful health impacts on the body. This research aimed to evaluate the interactive effects of swimming HIIT and resveratrol consumption on SIRTs 3 & 4, NAD+/NADH, AMPK and SOD2 expression in aged rats.

**Materials and Methods:**

In total, forty-five old male albino rats (Wistar) with the age of twenty months were allocated into 5 groups randomly. Control group (Ctrl), Swimming HIIT group (Ex: Exercise), Swimming HIIT with Resveratrol consumption group (R+Ex), Resveratrol consumption group (R) and solvent of resveratrol consumption group (vehicle). R+Ex group accomplished the exercise and consumed resveratrol (10 mg/kg/day, gavage) for 6 weeks.

**Results:**

HIIT & resveratrol significantly increased NAD+/NADH, SOD 2 and AMPK in the aged rats. HIIT increased SIRT3, but resveratrol reduced it. As for SIRT4, HIIT decreased it, while resveratrol positively affected it.

**Conclusion:**

Resveratrol and HIIT, especially their combination, have anti-oxidant and anti-aging effects on the hippocampus of old rats.

## Introduction

Aging is a gradual breakdown in the structure and organism of the body that occurs over time. The aging process is a complex phenomenon and elevates the risk of disability and death by gradually reducing the physiological function of the body and accumulating various harmful changes in the cells and tissues. Aging alters the structure and function of various organs of the body, such as reduced vision, hearing loss, slowness of movement, decreased muscle strength, reduced age-related neurons, impaired memory and perception, impaired orientation and decreased capacity and efficiency of the pulmonary and cardiovascular systems (1-4). Mitochondrial dysfunction is strongly involved in the aging process (5). It also has severe cellular consequences and is associated with aging and neurological disorders in humans (6). Sirtuins are a genes that have anti-aging function and establishes a link between aging and the metabolisms of body via mitochondria performance (7). Three of these sirtuins are dynamic in mitochondria: SIRT3, SIRT4 and SIRT5. SIRT3-dependent mitochondrial adjustments may be a principle pathway in postponing aging processes in mammals (8); therefore, SIRT3 seems to be a adapting molecule that modulates mitochondrial homeostasis via modulating target metabolic protein acetylation such as those that are a part of the endogenous portion of the antioxidant defense system (9). Besides, SIRT4 has been demonstrated to adjust oxidation of fatty acids and mitochondrial gene expression (10). However, based on the goals of SIRT4, it can contribute to ROS (reactive oxygen species) homeostasis by affecting SOD2 (superoxide dismutase 2) (11). But the question is whether SIRT4 also affects SOD2 in the brain or not. Although regulating energy flow across the glycolysis pathway seems to be one of the main functions of SIRT5, the targets of SIRT5 are not well understood. The relationship between structural and functional changes in SIRT5 has not been investigated; however, a decrease in SIRT5 expression in the aging process of the rat brain has been observed (9). On the other hand, NAD ^+^ (nicotinamide adenine dinucleotide) biology is among the most active research areas of the aging process (11). Recent advances have shown that NAD ^+^ reduction contributes to cellular and mitochondrial degeneration during the aging process (12). The NAD ^+^ / NADH ratio is complexly tied to cellular and mitochondrial metabolism. Previous studies have demonstrated training and induction of synaptic activity by SIRT3 expression in the hippocampus regulate mitochondrial protein acetylation and enhance neural defense against to oxidative stress and apoptosis (13). In the case of SIRT4 and exercise, one study found that eliminating SIRT4 decreased Malonyl CoA and increased exercise capacity, and suggested that SIRT4 may act in rearrangements during training (14). 

Resveratrol has useful anti-aging impacts, such as cardiovascular protection, anti-cancer, anti-diabetic, neuroprotective and anti-inflammatory (15, 16). It also prevents the high-excitation of neurons by prohibiting the activity of excitatory postsynaptic potential, therefore diminishing neuronal harm. Resveratrol can ameliorate cognitive functions in the patients with cognition disorders (17-19). Effects of resveratrol on AMPK (AMP activated protein kinase)-SIRT1-PGC-1α (peroxisome proliferator-activated receptor gamma coactivator-1 alpha) pathway have also been reported (20, 21). However, no research has been conducted on the effects of resveratrol on the hippocampal tissue and mitochondrial sirtuins, while the effect of resveratrol on SIRT4 is still under investigation (22). 

A review of the literature revealed that most of the limited available studies about the effect of exercise on the amount of these proteins have focused on various sports activities, including running on a treadmill or spinning wheel, while swimming is neglected. 

Thus, it seems necessary to study the effect of combined exercise intervention and resveratrol. The results of previous studies have shown that the factors involved in the balance of ROS lead to antioxidant defense and contribute to the regulation of cellular and mitochondrial homeostasis, which promotes optimal mitochondrial function and, ultimately, ensures cell survival. Consequently, we decided to investigate the impacts of HIIT swimming and resveratrol administration on sirtuins (SIRTs 3 & 4), NAD ^+^ / NADH, AMPK and superoxide dismutase 2 in the hippocampus of old rats.

## Materials and Methods

***Aging assessment***

To assess aging, 60 male 10-month-old Wistar rats weighing 300-350 g were kept in cages for 10 months and reached the weight range of 350-450 g, at which point cognitive decline was found to be a prominent indicator. To detect the onset of the aging process, for cognitive assessment (as an indicator of aging) and its non-interference with the exercise protocol, the rats were tested by the novel object recognition test before starting the study. Moreover, to ensure lack of motor disorder in old rats, the open field test was performed. Finally, 45 20-month-old male Wistar rats with the weight range of 350-450 g, the novel object recognition test of which was negative (had reduced cognitive memory) and had no movement disorders were included in the study. We reported these data in our previous study (23).

***Animals***

All the laboratory actions and interventions were confirmed by Ethics Committee of Kerman Neuroscience Research Center. Attempts were made to lessen the suffering of the animals at all steps of the research (ethics code: KNRC/24-96/EC). Forty-five old male albino (Wistar) rats (Twenty months old, weighing 350-450 g) were utilized for the present research. The rats (n=9 per group) were housed in groups of five with free access to water and food. They were caged under normal temperature (23±1 ^°^C) and 12 hr light-dark cycle (lights on 07:00–19:00 h). The forty-five old male rats were randomly allocated to the following groups: 1) Control group (Ctrl): with no training and treatment, 2) Swimming HIIT (Ex: Exercise): performed exercise, 3) Resveratrol administration (R): consumed resveratrol, 4) Swimming HIIT with resveratrol administration (R+Ex): consumed resveratrol with exercise and 5) Solvent of resveratrol administration (Vehicle): consumed methylcellulose. Finally, 48 hr after the last exercise session, the rats were anesthetized in a desiccator attached to a carbon dioxide capsule and sacrificed.

***Exercise protocol***

The animals in the Ex group performed the HIIT swimming exercise, which consisted of 14 20-sec swimming sessions with a 10-sec break between every two sessions. This exercise program was performed for six weeks (three days a week, one day in between). In the periodic load exercise applied in the first week, the weight was 9% of each rat’s body weight and 1% was added to it every week (23, 24). They swam at 14% of their body weight, which was attached to the root of their tails (Table 1-2) (25-27). The rats in the R+Ex group performed the swimming HIIT exercise with a 1% carboxymethylcellulose-soluble resveratrol supplement. The HIIT swimming exercise (similar to the Ex group), in the evening (the best training time in the controlled and normal performance rhythm of the animals), was performed under red light (to diminishing stress) (28). Each rat’s swimming speed and distance moved were recorded by a smart video tracking device (Noldus Ethovision System 7) connected to a computer screen. The rats received 10 ml of 1% oral carboxymethylcellulose solution by gavage on the daily basis.

***Assessing the intensity of exercise***

To evaluate the intensity of training in the first and third sessions of each week, the blood lactate of the rats of two training groups (R+Ex and Ex) was taken immediately at the end of the last (14^th^) turn of the tail end vessel using a Scout portable lactometer (model EKF, Germany) (27, 29, 30).

***NAD*** ^+^ ***/ NADH tissue assay***

The amount of NAD ^+^ protein was measured by a NAD^+^/ NADH assay kit (ab65348, Abcam Company, USA) using a BioTek spectrophotometer (USA) according to the kit instructions.

***Western blot analysis***

 After collecting all the samples, they were washed in the PBS solution. Homogenization was performed in RIPA lysis buffer solution and inhibitors of aprotinin (A1153sigma), leupeptin (L2023 Sigma), phenylmethylsulfonyl fluoride (P7626sigma) and sodium orthovanadate (S76). A sonic homogenizer performed all the steps of homogenization on ice and maintained cold conditions. The next step was tissue homogenization (centrifuged at 4000 ^°^C at 13000 round per minute for 20 min) and the supernatant was detachment. Bradford & bovine serum albumin determined the total protein concentration in the samples and the standard was set. After adjusting the concentrations, 40 μg of protein from each fragment was mixed in a buffer at the ratio of 1:5 samples. They were then centrifuged at 14,000 rpm for 4 min at 4 ^°^C (Eppendorf, USA). The supernatant was transferred to clean microtubules. Using the standard curve and the Bradford method, the appropriate protein density of the samples was calculated. A 4% sample buffer was added to the added material and heated at 95 ^°^C for 5 min to denature the proteins in equal parts. Equal amounts of protein were isolated with 12.5% ​​SDS-PAGE polyacrylamide gel. The vertical electrophoresis step was performed by a special tank (Bio-Rad) with 80-100 v. The proteins isolated in the gel were then transferred to the PVDF membrane (0.45 mm sc-3723) by a 220 mA current for 80 min. The membranes were immersed in a 5% blocking solution for 2 hr. They were then diluted in primary antibodies (anti-SIRT4 antibody ab124521, SIRT3 antibody (F-10) sc-365175, SIRT5 antibody (G-2) sc-271635, SOD-2 (E-10) sc-137254) and β-actin (monoclonal-A2228) with 2.5 SK of skim milk / TBS-T-Tween 20 in 1/1000. The incubation ratio at 18 ^°^C lasted for 18 hr. In the next step, the membranes were washed in a washing solution (TBS-T-Tween 20) in triplicate for 5 min each time. They were then incubated for 1 h in the secondary antibody (rabbit anti-mouse IgG-HRP: sc-358914) with 2.5% skim milk / TBS-T-Tween20 1/2000 at the room atmosphere. After being washed, the membranes were incubated with ECL (GERPN2232) and the light emitted from the luminescence reaction was recorded on a Geldoc system. ImageJ software was used to check the density of the films.

***Statistical analysis***

All Data were showed as mean±standard deviation (SD). The tested groups’ diversities were tested by one-way analysis of variance (ANOVA) followed by a post-hoc test (Tukey). *P-values*<0.05 were considered statistically significant. Statistical tests were performed in SPSS (IBM SPSS Statistics for Windows, version 22.0) (31, 32).

## Results

***The Eﬀect of HIIT and resveratrol on the animals’ weight***

Our results showed that the weight of the animals in all the groups did not change significantly in the first, third and sixth weeks. In other words, Ex and R had no effect on the animals’ weight (Table 3).

***The Eﬀects of HIIT and resveratrol on the swimming speed, swimming distance moved in each bout and total swimming distance moved per section***

Swimming speed, swimming distance moved in each bout and total swimming distance moved in each section were recorded by a video camera and analyzed via Ethovision 7 software. The results demonstrated that all the parameters between Ex and Ex+R groups in the 3rd week were remarkably higher than the 1st week (*P<*0.05). Furthermore, in the sixth week, the parameters were significantly higher than the fourth week (*P<*0.05; Table 2).

***The eﬀect of HIIT and resveratrol on the blood lactate***

In the first, third and sixth weeks, the samples of blood were compiled from the vein of tail of the animals and, using a lactometer, the blood lactate data were calculated. The blood lactate in the third week in R+Ex was considerably more than the 1st week (*P<*0.05). In the sixth week in the R+Ex group, the blood lactate was more than the first week and, in Ex, it was significantly less than the first week (*P<*0.05; Table 1).

***The eﬀect of HIIT and resveratrol on SIRT3 and SIRT4 expressions***

**Figure 1:**
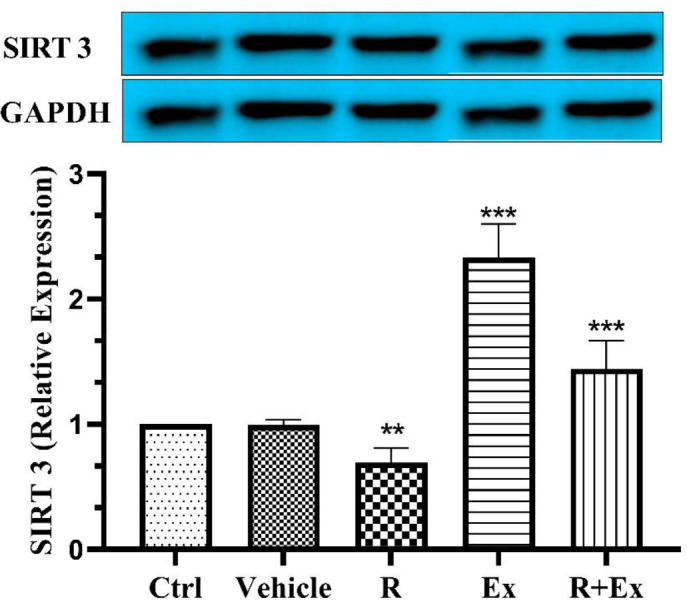
The effect of HIIT and Resveratrol on SIRT 3 expression in hippocampus of aged rats. There was a significant increase in SIRT 3 expression in Ex and R+Ex groups and a reduced expression in R group compared to Ctrl group. Mean±SD , (**) *P<*0.01 & (***) *P<*0.001

**Figure 2:**
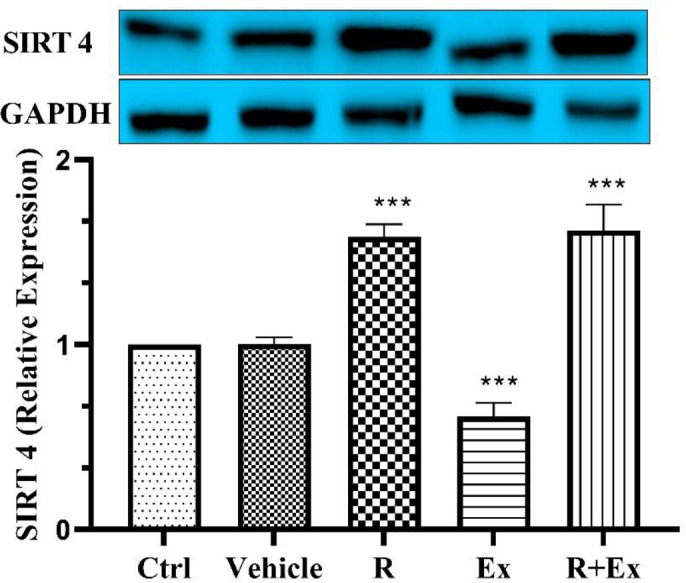
The effect of HIIT and Resveratrol on SIRT 4 expression in hippocampus of aged rats. There was a significant increase in SIRT 4 expression in R and R+Ex groups and a reduced expression in Ex group compared to Ctrl group. Mean±SD , (***) *P<*0.001

Our results showed that 6 weeks of resveratrol consumption significantly decreased the SIRT3 protein level (Figure 1, *P<*0.01) and significantly increased the SIRT4 protein level (Figure 2, *P<*0.001) in the R group than the control group. Swimming HIIT alone in the Ex group increased SIRT3 protein level (Figure 1, *P<*0.001) and decreased SIRT4 protein level (Figure 2, *P<*0.001) significantly in comparison with the control group. In the R+Ex group that received resveratrol with exercise, both SIRT3 and SIRT4 protein levels were remarkably more than the control group (Figure 1, *P<*0.001 & Figure 2, *P<*0.001).

***The eﬀect of HIIT and resveratrol on the SOD 2 expression***

**Figure 3:**
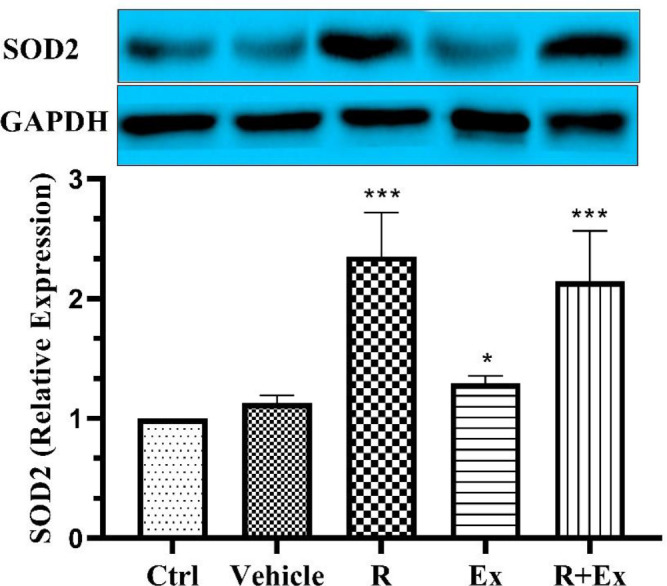
The effect of HIIT and Resveratrol on SOD 2 expression in hippocampus of aged rats. There was a significant increase in SOD 2 expression in R and R+Ex and Ex groups compared to Ctrl group. Mean±SD , (*) *P<*0.05 , (***) *P<*0.001

Our findings revealed that six weeks of resveratrol consumption significantly increased the SOD 2 protein level in the R group in comparison with the control group (Figure 3; *P<*0.001). Swimming HIIT alone in the Ex group significantly increased the SOD2 protein level in comparison with the control group (Figure 3; *P<*0.05). In the R+Ex group that received resveratrol with exercise, the SOD 2 protein level was considerably more than the control group (Figure 3; *P<*0.001).

***The eﬀect of HIIT and resveratrol on the AMPK expression***

**Figure 4:**
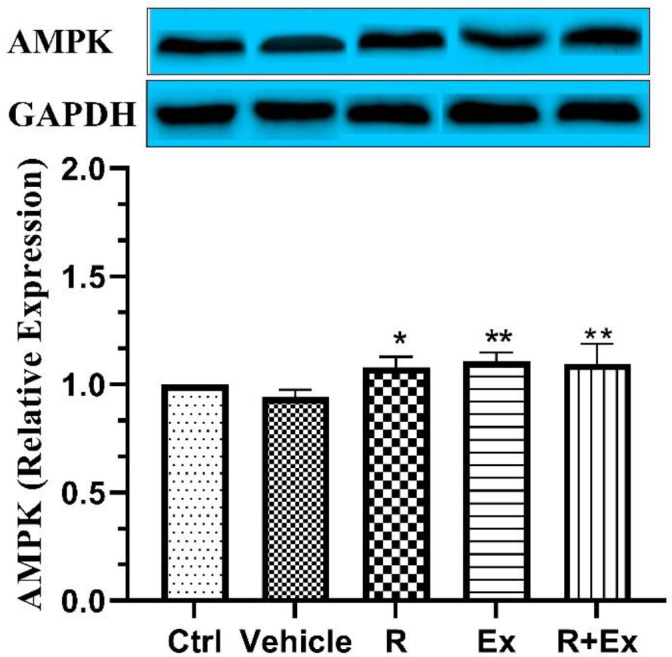
The effect of HIIT and Resveratrol on AMPK expression in hippocampus of aged rats. There was a significant increase in AMPK expression in R and R+Ex and Ex groups compared to Ctrl group. Mean±SD , (*) *P<*0.05 , (**) *P<*0.01

Our results disclosed that six weeks of resveratrol consumption significantly increased the AMPK protein level in the R group in comparison with the control group (Figure 4 ; *P<*0.05). Swimming HIIT alone in the Ex group significantly raised the AMPK protein level in comparison with the control group (Figure 4; *P<*0.01). In the R+Ex group that received resveratrol with exercise, the AMPK protein level was remarkably more than the control group (Figure 4; *P<*0.01).

***The eﬀect of HIIT and resveratrol on the NAD*** ^+^ ***/NADH ratio***

**Figure 5:**
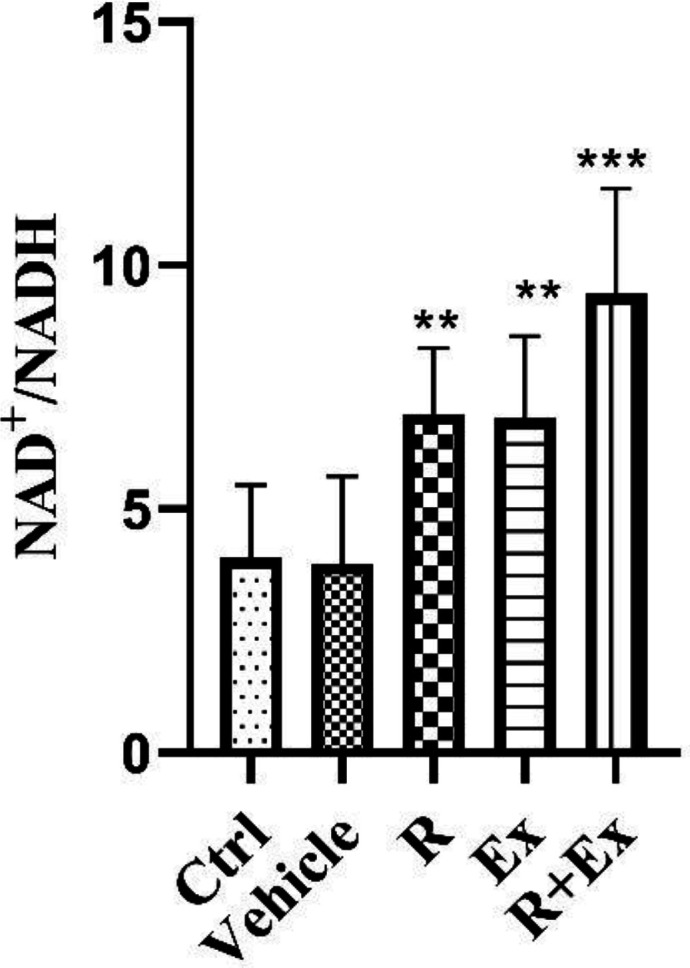
The effect of HIIT and Resveratrol on NAD^+^/NADH in hippocampus of aged rats. There was a significant increase in NAD^+^/NADH in R and R+Ex and Ex groups compared to Ctrl group. Mean±SD, (**) *P<*0.01 , (***) *P<*0.001

Our findings showed that six weeks of resveratrol consumption considerably elevated the NAD^+^/NADH ratio in the R group in comparison with the control group (Figure 5; *P<*0.01). Swimming HIIT alone in the Ex group increased NAD^+^/NADH ratio (Figure 5, *P<*0.01) in comparison with the control group. In the R+Ex group that received resveratrol with exercise, the NAD^+^/NADH ratio was remarkably more than the control group (Figure 5, *P<*0.001)

**Table 1:** Average changes in blood lactate of aged rats in swimming high-intensity interval trainin in 1^st^, 3^rd^ and 6^th^ weeks (Mean±SD)

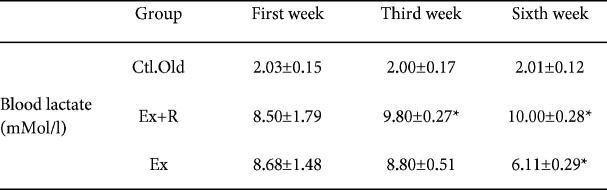

The amount of blood lactate in 6^th^ weeks in the aged rats of Ex group declined compared to the first week (*P=*0.0001). In contrast, the amount of blood lactate in 3^rd^ and 6^th^ weeks in aged rats of R+Ex group increased (*P=*0.0001). * Significant difference compared to the first week

Ex:Exercis , R:Resveratrol

**Table 2:** Average speed and distance in swimming high-intensity interval trainin of aged rat in 1^st^, 3^th^ and 6^th^ weeks. (Mean±SD)

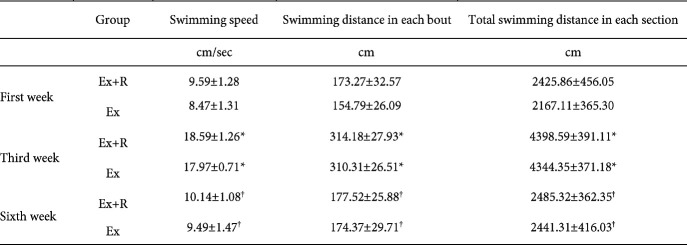

The swimming speed, swimming distance in each bout and total swimming distance in each section in 4^th^ weeks in the aged rats of Ex and R+Ex groups Significant increased compared to the 1^st^ week (*P=*0.0001). In contrast, the swimming speed, swimming distance in each bout and total swimming distance in each section in 6^th^ weeks in aged rats of Ex and R+Ex Significantly declined compared to the 4^th^ week (*P=*0.0001). * Significant difference compared to the first week and †: Significant difference compared to the fourth week) Ex: Exercise, R: Resveratrol

**Table 3:** The variation of weight in different groups in 1^st^, 3^th^ and 6^th^ weeks (Mean±SD)

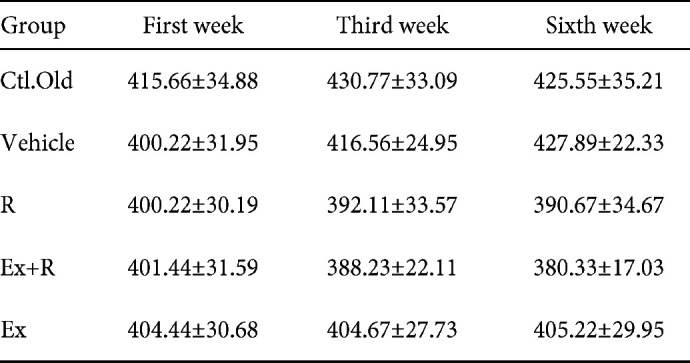

Ex:Exercis , R:Resveratrol

HIIT: High Intensity Interval Training , R: Resveratrol , Ctrl: Control , Ex: Exercise

HIIT: High Intensity Interval Training , R: Resveratrol , Ctrl: Control , Ex: Exercise

HIIT: High Intensity Interval Training , R: Resveratrol , Ctrl: Control , Ex: Exercise

HIIT: High Intensity Interval Training , R: Resveratrol , Ctrl: Control , Ex: Exercise

HIIT: High Intensity Interval Training , R: Resveratrol , Ctrl: Control , Ex: Exercise

## Discussion

We evaluated the impacts of resveratrol and swimming HIIT exercise on SIRT3, SIRT4, SOD 2 and AMPK protein level expression and NAD/NADH ratio in aged rats.

Our findings revealed that resveratrol alone, HIIT alone and their combination in old rats increased NAD^+^/NADH ratio compared to control old rats.

Resveratrol increases NADH dehydrogenase activities accompanied by a stimulation of NADH oxidation and can raise the NAD^+^/NADH ratio (33). Grant *et al*. showed that resveratrol raises intracellular NAD^+^ contents via the enhancing of the NAD^+^ synthetic enzyme nicotinamide mononucleotide adenylyl transferase (34). Resveratrol can directly activate SIRT1 by increasing the mitochondrial NAD^+^/NADH ratio (35). These observations agree with our results.

In the EDL (Extensor digitorum longus) and soleus muscles of the rat, tetanic or twitch contractions increase NAD^+^ contents (as tested by reduced NADH fluorescence) during contraction (36). Increased ATP demand during exercise raises the free cytosolic/nuclear and mitochondrial NAD content as well as NAD/NADH ratio, which gives an elevated substrate for the NAD-consuming enzymes (in purple), SIRT1, SIRT3, PARP1 and PARP2. Exercise also reduces the availability of NADH (37). The NAD^+^/NADH ratio and its individual subcellular levels reflect the overall energy and redox status of the cell (38). During high-intensity exercise, NADH is produced in glycolysis and transported to the mitochondria for oxidation or, with pyruvate, it is processed by lactate dehydrogenase (LDH) to NAD^+^ and lactate. There are different opinions and research rationales in studies about the direct relationship between cellular NAD^+^/NADH changes and exercise volume and intensity (37, 38). The calculated cytosolic NAD^+^/NADH ratio is reduced after 60 min of cycling exercise at ~62%VO_ 2max or after the Wingate Test (39, 40). Nevertheless, Phillips *et al*. showed that the calculated ratio was reduced after 15 min and, subsequently, returned to resting levels by 90 min of exercise at 59%VO_ 2max (41). de Guia *et al*. revealed that aerobic and resistance exercise training reversed the age-dependent decline in NAD^+^ salvage capacity in human skeletal muscles (42). Other studies have concluded that HIIT exercise can increase the NAD^+^/NADH ratio (43, 44). Our results also showed that HIIT exercise increased NAD/NADH ratio in old rats. However, it seems that changes in NAD/NADH ratio after exercise depend on factors such as the intensity, type and time of exercise. In this study, the combination of resveratrol and HIIT exercise also had additive effects on this ratio.

Our results disclosed that resveratrol alone, HIIT alone and their combination in old rats raised AMPK expression compared to control old rats.

AMPK has emerged as a key nutrient sensor with the ability to regulate whole-body metabolism. It is an evolutionarily conserved enzyme, the activity of which is triggered by increased AMP/ATP ratio that reflects the energy status of the cell. Resveratrol is an AMPK activator through affecting the mitochondrial metabolism (45).

Lan *et al*. showed that resveratrol activated AMPK through the activation of the SIRT1-LKB1-AMPK feedback loop. Resveratrol-induced activation of AMPK requires the presence of functional LKB1 (SIRT1-liver kinase B1) and resveratrol increases the LKB1 activity (46). Chiang *et al*. reported that resveratrol can have a neuroprotective role in the Alzheimer’s disease via the AMPK activation (47). Pineda-Ramírez demonstrated that resveratrol activated neuronal autophagy through AMPK in the ischemic brain (48). Furthermore, Li *et al*. showed that resveratrol alleviated THEs brain injury following subarachnoid hemorrhage via the activation of AMPK/SIRT1/autophagy signaling pathway (49).

The great energy demand of HIIT causes in noticeable elevating in cellular AMP/ATP and ADP/ATP ratios which can up regulate AMPK, one of the most important energy sensors of the cell (38). Several studies have shown that AMPK phosphorylation in the skeletal muscles positively correlates with exercise intensity (50, 51) and duration (52). Gibala *et al*. reported that brief intense interval exercise activated AMPK and p38 MAPK signaling and increased the expression of PGC-1alpha in the human skeletal muscles (53).

Based on our results, resveratrol alone, HIIT alone and their combination in old rats increased SOD2 expression compared to control old rats.

Resveratrol can boosts the PI3K/Akt signaling pathways and the augmented Akt, finally, inactivated GSK-3β and stabilized β-catenin. The stabilized β-catenin translocates into the nuclei, binds to the TCF family transcription factors or FOXO, and induces the expression of target genes, including SOD2. SOD2 proteins suppress ROS in the mitochondria due to glutathione reduction (54). Resveratrol has also been shown to exert beneficial neuroprotective effects through its antioxidant capacity (55, 56). Mathieu *et al*. showed that resveratrol attenuated oxidative stress in mitochondrial complex I deficiency via a direct scavenging role and inducing endogenous anti-oxidative enzymes such as SOD2 (55).

 Bogdanis G.C reported that three weeks of HIIT in humans improved antioxidant capacity in their plasma (56). They also demonstrated that short-term high-intensity interval exercise training attenuated oxidative stress responses and improved the antioxidant status in healthy humans (57).

Based on our findings, HIIT alone decreased SIRT4 expression, but resveratrol alone and their combination with HIIT in old rats increased SIRT4 expression compared to control old rats.

Almost all articles have reported the stimulatory role of resveratrol on the expression and amount of SIRT4 (22, 58, 59). Our results also showed that resveratrol alone and in combination with exercise had potentiating effects on the expression of SIRT4.

NAD+-dependent SIRT4 has been showed to be a main modulator of antioxidant defense mechanisms and metabolic enzymes in mitochondria. It also act in regulating the mitochondrial metabolism due to training (60). Unlike other sirtuins, the apprehension of the performances and goals of SIRT4 in training is still so restricted. Owing to its complicated impacts on mitochondrial adaptive capacity and mitochondrial energy metabolism to training, it is so probable that SIRT4 can be regulated by training (60). Hart *et al*. showed that a twelve-week treadmill exercise considerably diminished SIRT4 content in gastrocnemius muscle of the rats selectively bred as low-capacity runners (LCRs) and high-capacity runners (HCRs), respectively, proposing that diminished SIRT4 content caused in elevated free fatty acid utilization as a main factor for increasing endurance capacity (61, 62). These findings also show that the downregulating impacts of exercise on SIRT4 level could have useful impacts on glucose homeostasis because SIRT4 is demonstrated to be participated in the evolution of insulin resistance (63). Laurent *et al*. demonstrated that SIRT4 KO mice can run 20% longer distance and for a longer duration than control mice in response to graded, maximal treadmill challenge, which might be attributed to elevated enhanced exercise capacity , fatty acid oxidation and alleviated resistance to diet-induced obesity (14). Since the reduce of malonyl CoA can elevate fat oxidation in muscles after training (64), SIRT4 knockout resulted to a diminishing of malonyl coenzyme contents and elevate of training capacity, indicating that SIRT4 may participate to metabolic reprogramming during exercise (14). Karvinen *et al*. disclosed that the level of SITR4 protein was unchanged in the rat skeletal muscle after 1 year of wheel running (65). However, a research of mountaineers revealed that the SIRT4 mRNA expression in the skeletal muscle increased after 5 weeks of exposure to high altitude which included climbing peaks over 8,000 m, proposing that SIRT4 is participated in the changes in fatty acid metabolism at high altitude and with training (66, 67). In general, the studies revealed that NAD^+^-dependent SIRT4 is so susceptible to metabolic stresses and may be regulated by training, although there have been debates about the expression of SIRT4 depending on the cell type (60). Our findings indicated that swimming HIIT decreased SIRT4 expression compared to old rats. It seems that some factors such as the type, duration and intensity of exercise can have various impacts on the SIRT4 expression. Furthermore, the type of tissue (muscle, adipose tissue, liver or brain tissues) is a major determinant in SIRT4 expression in response to exercise.

Our results revealed that resveratrol alone decreased SIRT3 expression, but HIIT alone and their combination with resveratrol in old rats elevated SIRT3 expression compared to control old rats.

Training makes cellular metabolic tension, which affects the sirtuins (44). Palacios *et al*. showed that mouse skeletal muscle SIRT3 responded actively to 6 weeks of wheel running training to modulate the downstream molecular response. They found that training elevated the SIRT3 protein level (68). Hokari *et al*. approved the elevate in the SIRT3 level of skeletal muscle in rats after four weeks of wheel running training or treadmill exercise; moreover, they demonstrated that the SIRT3 level was decreased in the sedentary soleus muscle (69). Muñoz *et al*. demonstrated that physical exercise improved aging-related changes in SIRT3 in substantia nigra (70). Our results also indicated that HIIT increased SIRT3 expression.

As for resveratrol, many studies have demonstrated the stimulatory effect of resveratrol on SIRT3 (71-73). Our results showed that resveratrol alone reduced SIRT3 expression, which is contrary to other studies. However, in combination with exercise, it greatly increased the expression of SIRT3. To justify this difference, it may be possible to point to differences in factors such as the age of the animals, the tissue studied and the dose or concentration of resveratrol.

In the case of changes in rat’s weight it’s necessary to say that Ctrl and vehicle groups which had no any intervention, in rat’s weight in sixth week compare to first week was increased but was not statistically significant. It seems the metabolic condition of rats was the cause of this matter. the normal metabolic condition is one of main factors to regulate the metabolic balance which probably leads to no significant changes in Ctrl and vehicle groups rat’s weight. Moreover, the rat’s weight in R+EX and R groups was decreased but was not statistically significant. Considering table 1 decreased rat’s weight in group R+EX has been more than group R that shows swimming HIIT with resveratrol supplementation leads to more weight loss. In addition to creating adaptations resulting from swimming HIIT and resveratrol, likely opening the metabolic window after exercise leaded to normal weight loss in intervention groups. But swimming HIIT alone (EX group) couldn’t make changes in rat’s weight. On the other hand, a meta-analysis study showed resveratrol can decrease body weight and showed that resveratrol consumption remarkably diminished BMI, fat mass and weight and considerably elevated lean mass (74). When it comes to exercise and weight loss it must be certain that which one is lost: fat mass or muscle mass. In the present study Due to the nature of swimming HIIT and the intensity was used probably fat mass has been reduced and muscle mass increased. It seems this is the cause of consistency of the rat’s weight in EX group. 

In a previous study, we reported that aging has adverse behavioral impacts on animals, and resveratrol and HIIT could ameliorate these negative effects (23). In general, some researches have suggested that aging has adverse impacts on memory and the hippocampus (75, 76). Aging disrupts rats’ function in some hippocampal-dependent cognitive tests including the Morris water maze (MWM) (77, 78), Barnes maze (79) and radial arm water maze (RAWM) (80).

In short, sirtuins must be noticed as some of the major goals in curing cognitive disorders. Resveratrol showed the capability to improve cognition by modulating SIRTs through AMPK and several other molecular pathways. Its features such as anti-inflammatory , anti-oxidative and anti-apoptotic regulation and autophagic, as well as its potency to ameliorate cerebral blood flow and augment synaptic plasticity (72). Qiu *et al*. demonstrated that the dysregulation of BDNF(Brain derived neurotrophic factor)/TrkB signaling mediated by NMDAR (N-methyl-D-aspartate receptor)/Ca 2+/calpain might participate to memory impairment in old mice (81). Belviranl *et al*. found that exercise protected against aging-induced memory impairment via activating the hippocampal PGC-1α/FNDC5/BDNF pathway (75).

However, it seems that more electrophysiological or molecular investigations are needed to clarify the issue in future studies.

## Conclusion

Our results suggested that both resveratrol and HIIT, especially their combination, had anti-oxidant and anti-aging effects in old rats. It seems that their combination could strongly reverse the negative consequences of aging.

## Authors’ Contributions

AM, FD, AG Study design and data analysis, writing the paper draft; MA Correcting the draft, supervising the experiments; MAR Writing the drafts, performing the experiment; MAB Writing the drafts, performing the experiment; KHE performing the experiment.

## Conflicts of Interest

The authors declare there are no conflicts of interest.
